# Memory formation and long-term maintenance of IL-7Rα^+^ ILC1s via a lymph node-liver axis

**DOI:** 10.1038/s41467-018-07405-5

**Published:** 2018-11-19

**Authors:** Xianwei Wang, Hui Peng, Jingjing Cong, Xuefu Wang, Zhexiong Lian, Haiming Wei, Rui Sun, Zhigang Tian

**Affiliations:** 1 Division of Molecular Medicine, Hefei National Laboratory for Physical Sciences at Microscale, the CAS Key Laboratory of Innate Immunity and Chronic Disease, School of Life Sciences, University of Science and Technology of China, Hefei, 230027 Anhui China; 2 Institue of Immunology, University of Science and Technology of China, Hefei, 230027 Anhui China

## Abstract

Natural killer (NK) cells are reported to have immunological memory, with CD49a^+^ liver-resident NK cells shown to confer hapten-specific memory responses, but how this memory is induced or maintained is unclear. Here we show that memory type I innate lymphoid cells (ILC1s), which express IL-7Rα, are generated in the lymph nodes (LNs) and require IL-7R signaling to maintain their longevity in the liver. Hapten sensitization initiates CXCR3-dependent recruitment of IL-7Rα^+^ ILC1s into skin-draining LNs, where they are primed and acquire hapten-specific memory potential. Memory IL-7Rα^+^ ILC1s then exit draining LNs and are preferentially recruited, via CXCR6, to reside in the liver. Moreover, long-term blockade of IL-7R signaling significantly reduces ILC1-mediated memory responses. Thus, our results identify a memory IL-7Rα^+^ ILC1 population and reveal a LN-liver axis that is essential for ILC1 memory generation and long-term maintenance.

## Abstract (web summary)

Natural killer cells may respond better on second antigen encounters, but how this memory is induced or maintained in vivo is not clear. Here the authors show that memory NK cells expressing interleukin-7 (IL-7) receptor are induced in the lymph nodes but later recruited to liver for long term, IL-7 dependent survival and memory maintenance.

## Introduction

Innate lymphoid cells (ILCs) are a heterogeneous family of innate immune cells that are important for host defense and homeostasis^1–4^. Although ILCs belong to the innate immune system, accumulating evidence indicates that they also have adaptive immune features. Evidence has emerged that group 1 ILCs, consisting of conventional natural killer (cNK) cells and ILC1s, can generate long-term memory responses against haptens^5–7^, mouse cytomegalovirus (MCMV)^8,9^, and cytokine stimulation^10,11^. Moreover, NK cells that recall human cytomegalovirus^12,13^, human hantavirus^14^, and simian immunodeficiency virus^15^ have also been described in humans and rhesus macaques. Additionally, group 2 ILCs (ILC2s) have recently been demonstrated to possess memory-like properties in allergen-induced lung inflammation^16^. Overall, studies of ILC memory function have become increasingly important in the field of ILC research.

The first evidence supporting antigen-specific ILC memory came from studies by von Andrian and colleagues. They reported that bulk liver NK cells (now also referred to as group 1 ILCs), but not splenic NK cells, could induce hapten-specific skin contact hypersensitivity (CHS) responses, independent of T and B cells; thus the concept of NK cell memory was proposed^7^. A follow-up study demonstrated that the chemokine receptor, C-X-C chemokine motif receptor 6 (CXCR6), is critical for liver NK cell memory in CHS models^5^. Recently, we demonstrated that liver NK cells are a heterogeneous population, composed of CD49a^−^ cNK cells and CD49a^+^ liver-resident NK (LrNK) cells, the latter of which express high levels of CXCR6 and can confer hapten-specific CHS memory responses^6,17^. Although memory group 1 ILCs have not been described in human allergic contact dermatitis (ACD), human CD3^−^CD56^high^CD16^−^ NK cells accumulate in the skin of patients with ACD^18^, suggesting the importance of group 1 ILCs in human allergic skin inflammation. Despite such progress, the mechanisms underlying the formation and long-term maintenance of liver memory group 1 ILCs remain largely unknown.

Hapten-specific adaptive lymphocytes are primed in skin-draining lymph nodes (LNs) after hapten application to the skin; however, whether group 1 ILC-mediated memory responses occur in processes similar to those of T cells remains unknown. A unique NK subset, characterized by expression of CD127 (interleukin (IL)-7Rα), is present in the thymus and LNs of mice and humans^19^ and has been classified as interferon (IFN)-γ- and tumor necrosis factor (TNF)-producing non-cytotoxic ILC1s^20^. LN IL-7Rα^+^ ILC1s develop separately via thymus-dependent and thymus-independent pathways, unlike bone marrow (BM)-derived cNK cells^21^. The view that group 1 ILCs promote T helper type 1 (Th1) polarization via secretion of IFN-γ in LNs is widely accepted^22^; however, the hapten, fluorescein isothiocyanate (FITC), which induces Th2 responses^23^, also recruits group 1 ILCs into LNs^24^. Interestingly, our study demonstrated that FITC also induces LrNK cell-mediated immunological memory responses^6^. Whether LN group 1 ILCs are involved in this process has not been established.

IL-7Rα is expressed mainly on T cells, pro-B cells, dendritic cells (DCs), and ILCs^1,25,26^. A dramatic loss of T cells, B cells, ILC2s, and ILC3s is observed in IL-7- or IL-7Rα-deficient mice, whereas ILC1s and cNK cells are not affected^25–29^. IL-7R signaling contributes to sustaining the expression of the anti-apoptotic factors, BCL-2 and myeloid cell leukemia sequence 1, which promote the survival of memory T cells^30,31^. IL-7 can also induce triacylglyceride (TAG) synthesis, which fuels fatty acid oxidation (FAO) to maintain the longevity of memory CD8^+^ T cells^32^. Although IL-7R is not required for ILC1 development, whether IL-7R signaling plays a role in the longevity of hapten-induced memory ILC1s is unclear.

Here we identify memory IL-7Rα^+^ ILC1s in the LNs and liver and demonstrate the molecular mechanisms that occur during memory ILC1 formation and maintenance, unveiling a critical role for the LN–liver axis in ILC1 memory responses. We find that IL-7Rα^+^ ILC1s initially respond to haptens and acquire immunological memory in draining LNs and that LN-derived memory IL-7Rα^+^ ILC1s selectively reside in the liver, via CXCR6, maintaining their long-term homeostasis through IL-7R signaling. Our study sheds new light on the generation of ILC memory.

## Results

### Liver IL-7Rα^+^ ILC1s mediate hapten-specific memory responses

**Fig. 1:**
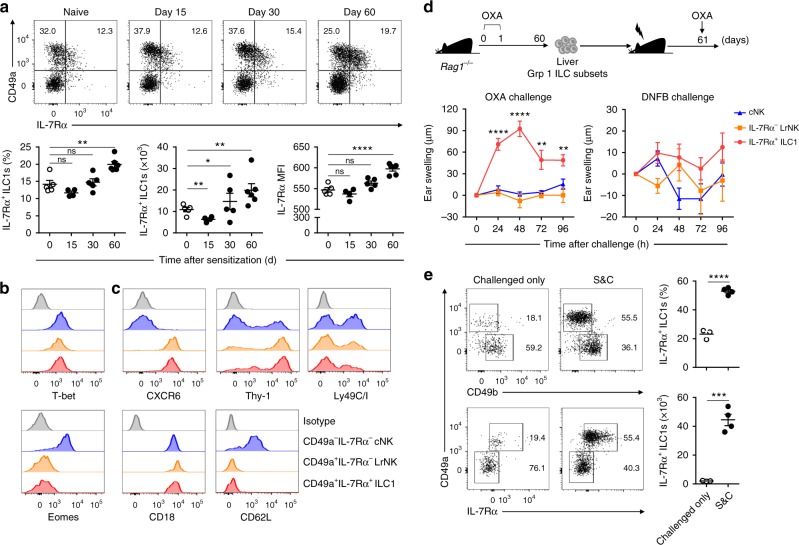
Liver IL-7Rα^+^ ILC1s confer hapten-specific CHS responses. **a** Representative dot plots (top panels) showing CD49a and IL-7Rα expression on liver CD3^−^NK1.1^+^NKp46^+^ cells at different time points after OXA sensitization in WT C57BL/6 (B6) mice. Statistical analysis of results (bottom panels) showing the percentages and absolute numbers of liver IL-7Rα^+^ ILC1s and mean fluorescent intensity (MFI) of IL-7Rα on liver ILC1s. Liver ILC1s were gated on CD3^−^NK1.1^+^NKp46^+^CD49a^+^IL-7Rα^+^. Data are representative of two independent experiments (*n* = 4–6 in each group). **b**, **c** Expression of the indicated transcription factors (**b**) or cell surface markers (**c**) by liver cNK cells (CD3^−^NK1.1^+^NKp46^+^CD49a^−^IL-7Rα^−^), IL-7Rα^−^ LrNK cells (CD3^−^NK1.1^+^NKp46^+^CD49a^+^ IL-7Rα^−^), and IL-7Rα^+^ ILC1s (CD3^−^NK1.1^+^NKp46^+^CD49a^+^ IL-7Rα^+^) from OXA-sensitized (day 60) WT B6 mice. Data are representative of at least three independent experiments (*n* = 3–5 in each experiment). **d** Ear swelling of WT B6 mice that received 5 × 10^4^ liver cNK cells, IL-7Rα^−^ LrNK cells, or IL-7Rα^+^ ILC1s from OXA-sensitized *Rag1*^−^^/^^−^ donors, followed by ear skin challenge with OXA or DNFB. Data are representative of two independent experiments (*n* = 4–5 in each group). **e** Representative dot plots (left panels) showing CD49a and IL-7Rα expression on CD45^+^NK1.1^+^NKp46^+^ cells in ear skin from OXA-challenged naive (challenged only) or sensitized (S&C) *Rag1*^−^^/^^−^ mice. Statistical analysis of results (right panels) showing the percentages and absolute numbers of ear skin IL-7Rα^+^ ILC1s. Data are pooled from two independent experiments (*n* = 3–4 in each group). Means ± SEM are shown. *P < 0.05, **P < 0.01, ***P < 0.001, ****P < 0.0001, two-tailed unpaired Student’s *t* test (**a**, **e**) and unpaired one-way ANOVA (**d**)

Previous studies have indicated that memory group 1 ILCs are restricted to the liver in CHS models^5–7,33^ and that a liver-resident CD49a^+^ subset is important in this process^6^. To obtain a more comprehensive understanding of memory group 1 ILC formation, we analyzed the phenotype of liver group 1 ILCs at different time points after sensitization with the hapten, oxazolone (OXA). Using CD49a, a well-established tissue-resident marker^6,34^, and IL-7Rα, a classic helper ILC marker^1–4^, we divided liver group 1 ILCs into three subsets: CD49a^−^IL-7Rα^−^ cNK cells, CD49a^+^IL-7Rα^−^ LrNK cells, and CD49a^+^IL-7Rα^+^ ILC1s. We found that the expression of IL-7Rα by liver IL-7Rα^+^ ILC1s was enhanced after hapten sensitization (Fig. 1a and Supplementary Fig. 1a). Moreover, the absolute number of IL-7Rα^+^ ILC1s, but not cNK cells or LrNK cells, exhibited a two-fold increase 60 days after sensitization (Fig. 1a and Supplementary Fig. 1b), while spleen group 1 ILC subsets exhibited no significant changes (Supplementary Fig. 1c). Further analysis revealed that there was a slight increase in liver IL-7Rα^+^ ILC1 numbers 96 h after sensitization (Supplementary Fig. 1d) and that the increase could last for at least 4 months (Supplementary Fig. 1e); however, expression of the proliferation marker, Ki67, in liver IL-7Rα^+^ ILC1s did not change, suggesting that the increase may be a consequence of cell migration, rather than local proliferation (Supplementary Fig. 1f). Similar to steady-state LrNK cells^35,36^, the IL-7Rα^+^ ILC1s that increased following hapten sensitization remained T-bet-positive and did not acquire Eomes expression (Fig. 1b). Compared with cNK cells, both IL-7Rα^+^ ILC1s and IL-7Rα^−^ LrNK cells constitutively expressed higher levels of CXCR6 and Thy-1 (Fig. 1c), which are associated with liver group 1 ILC-mediated CHS^5,7^, suggesting that IL-7Rα^+^ ILC1s may participate in hapten-induced memory responses. To test this hypothesis, we adoptively transferred OXA-sensitized liver IL-7Rα^+^ ILC1s, IL-7Rα^−^ LrNK cells, or cNK cells into naive mice, followed by challenge with OXA or dinitrofluorobenzene (DNFB). Interestingly, only IL-7Rα^+^ ILC1s, but not IL-7Rα^−^ LrNK or cNK cells, elicited significant CHS responses in a hapten-specific manner (Fig. 1d). Furthermore, after hapten challenge of sensitized *Rag1*^−^^/^^−^ mice, IL-7Rα^+^ ILC1s accumulated at the effector site, with an approximately 20-fold increase (Fig. 1e). These results demonstrate that IL-7Rα^+^ ILC1s are the cells responsible for hapten-specific memory responses in the CHS model.

### Haptens induce priming of IL-7Rα^+^ ILC1s in skin-draining LNs

**Fig. 2:**
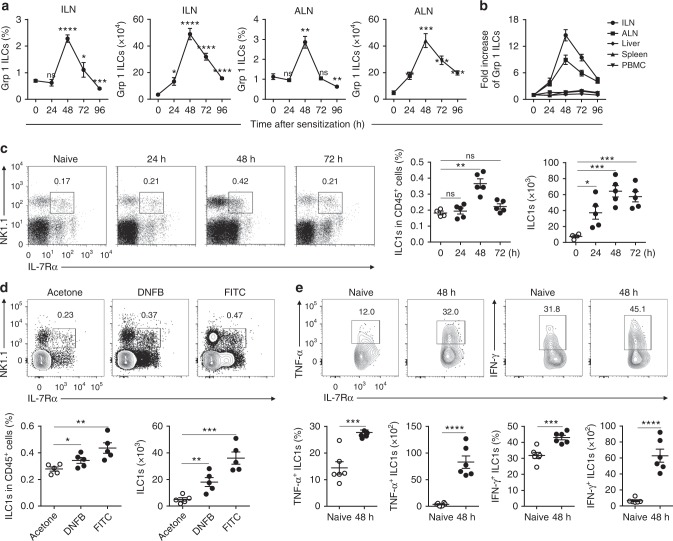
Haptens induce the accumulation and activation of IL-7Rα^+^ ILC1s in skin-draining LNs. **a**, **b** Percentages, absolute numbers, and fold increase in numbers of group 1 ILCs (CD3^−^NK1.1^+^NKp46^+^) from the indicated tissues of WT B6 mice after OXA sensitization. Data are pooled from four independent experiments (*n* = 4–6 in each group). **c** Representative dot plots (left panels) showing NK1.1 and IL-7Rα expression on ILN CD3^−^ cells from OXA-sensitized WT B6 mice. Numbers in dot plots represent the percentages of ILN IL-7Rα^+^ ILC1s cells among CD45^+^ cells. Statistical analysis of results (right panels) showing the percentages and absolute numbers of ILN IL-7Rα^+^ ILC1s. Data are representative of at least three independent experiments (*n* = 4–5 in each experiment). **d** Representative density plots (top panels) showing NK1.1 and IL-7Rα expression on ILN CD3^−^ cells from FITC- or DNFB-sensitized (48 h) WT B6 mice. Numbers in dot plots represent the percentages of ILN IL-7Rα^+^ ILC1s cells among CD45^+^ cells. Statistical analysis of results (bottom panels) showing the percentages and absolute numbers of ILN IL-7Rα^+^ ILC1s. Data are representative of two independent experiments (*n* = 5 in each experiment). **e** Representative density plots (top panels) showing expression of TNF and IFN-γ by ILN ILC1s (CD3^−^NK1.1^+^NKp46^+^IL-7Rα^+^) from naive or OXA-sensitized mice after stimulation of ILN cells with PMA/Ion (for TNF detection) or IL-12/IL-18 (for IFN-γ detection). Statistical analysis of results (bottom panels) showing the percentages and absolute numbers of TNF-producing or IFN-γ-producing ILN ILC1s. Data are representative of two independent experiments (*n* = 4–5 in each experiment). Means ± SEM are shown. *P < 0.05, **P < 0.01, ***P < 0.001, ****P < 0.0001, two-tailed unpaired Student’s *t* test

To identify the source of liver memory IL-7Rα^+^ ILC1s, we examined total group 1 ILCs in lymphoid and non-lymphoid tissues at different time points after OXA sensitization. In the early stage of sensitization (0–96 h), we observed that the percentage and absolute number of group 1 ILCs increased significantly in inguinal lymph nodes (ILNs) and axillary lymph nodes (ALNs) (Fig. 2a) but not in the liver or spleen (Fig. 2b). Detailed flow cytometric analyses revealed that group 1 ILCs in LNs primarily consisted of two subsets: NK1.1^+^NKp46^+^IL-7Rα^−^T-bet^+^Eomes^+^ cNK cells and NK1.1^+^NKp46^+^IL-7Rα^+^T-bet^+^Eomes^−^ ILC1s (Supplementary Fig. 2a–c). After skin sensitization with OXA, IL-7Rα^+^ ILC1s in abdomen skin-draining LNs initially increased both in frequency and in total number, with numbers peaking at 48 h (up to ten-fold increase), and then underwent contraction, evidenced by decreased cell numbers at 72 h (Fig. 2c and Supplementary Fig. 3a). No difference was observed in non-draining LNs (Supplementary Fig. 3b). Other haptens, such as DNFB and FITC, also induced accumulation of IL-7Rα^+^ ILC1s in skin-draining LNs (Fig. 2d and Supplementary Fig. 3c). Moreover, LN IL-7Rα^+^ ILC1s from hapten-sensitized mice produced significantly higher amounts of TNF and IFN-γ compared with those from naive mice (Fig. 2e and Supplementary Fig. 3d), confirming their activation state in response to haptens. Collectively, these results show that haptens can induce accumulation and priming of IL-7Rα^+^ ILC1s in skin-draining LNs.

### IL-7Rα^+^ ILC1 migration to skin-draining LNs requires CXCR3

**Fig. 3:**
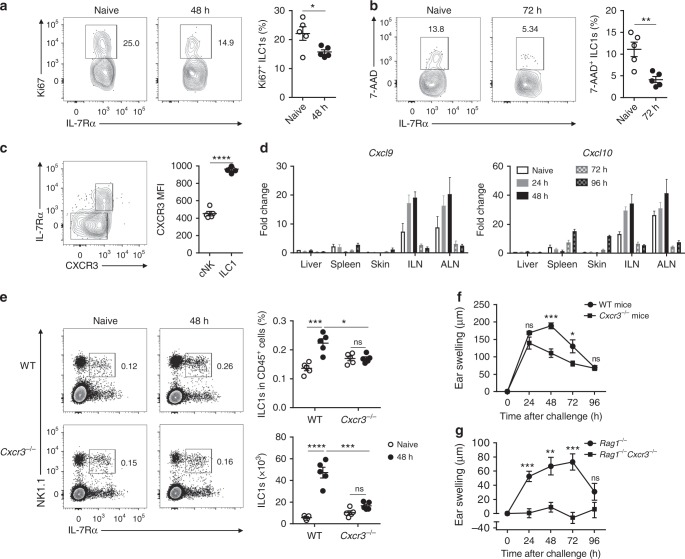
IL-7Rα^+^ ILC1 migration to skin-draining LNs is dependent on CXCR3. **a**, **b** Representative density plots showing Ki67 (**a**) or 7-AAD (**b**) staining on ILN CD3^−^NK1.1^+^IL-7Rα^+^ cells from OXA-sensitized (**a**: 48 h; **b**: 72 h) WT B6 mice. Statistical analysis of results showing the percentages of Ki67^+^ (**a**) or 7-AAD^+^ (**b**) cells among ILN ILC1s. Data are representative of two independent experiments (*n* = 5 in each group). **c** Representative dot plots (left panel) showing CXCR3 and IL-7Rα expression on CD3^−^NK1.1^+^NKp46^+^ cells from OXA-sensitized (48 h) B6 mice. Statistical analysis of results (right panel) showing MFI of CXCR3 on cNK cells and ILC1s. Data are representative of two independent experiments (*n* = 5 in each experiment). **d** mRNA expression of *Cxcl9* and *Cxcl10* in the indicated tissues after OXA sensitization. Data are pooled from two independent experiments (*n* = 3 in each group). **e** Representative density plots (left panels) showing CD49a and IL-7Rα expression on ILN CD3^−^ cells from OXA-sensitized (48 h) WT or *Cxcr3*^−^^*/*^^−^ mice (left panels). Numbers in dot plots represent the percentages of ILN IL-7Rα^+^ ILC1s among CD45^+^ cells. Statistical analysis of results (right panels) showing the percentages and absolute numbers of ILN IL-7Rα^+^ ILC1s. Data are representative of two experiments (*n* = 5 in each group). **f** Ear swelling of WT and *Cxcr3*^−^^*/*^^−^ mice sensitized (days 0, 1) and challenged (day 4) with OXA. Data are representative of two experiments (*n* = 5 in each group). **g** Ear swelling of *Rag1*^−^^*/*^^−^ and *Rag1*^−^^*/*^^−^*Cxcr3*^−^^*/*^^−^ mice sensitized (days 0, 1) and challenged (day 4) with OXA. Data are representative of two experiments (*n* = 5–6 in each group). Means ± SEM are shown. *P < 0.05, **P < 0.01, ***P < 0.001, ****P < 0.0001, two-tailed unpaired Student’s *t* test

Clonal expansion and apoptosis-induced contraction are important features of memory T cells and MCMV-specific memory Ly49H^+^ cNK cells^8,9^. In contrast, Ki67 expression in LN IL-7Rα^+^ ILC1s decreased slightly when cell numbers peaked in the CHS model (Fig. 3a and Supplementary Fig. 4a), suggesting that the increase of IL-7Rα^+^ ILC1s was not due to clonal expansion. During the contraction phase, LN IL-7Rα^+^ ILC1s did not exhibit increased apoptosis (7AAD^+^) (Fig. 3b and Supplementary Fig. 4b), suggesting that the contraction of IL-7Rα^+^ ILC1s could be due to their egress from skin-draining LNs, rather than a result of apoptosis. DC injection or viral infection can induce NK cell migration to draining LNs in a CXCR3-dependent fashion^22,37^. We thus examined CXCR3 expression in LN group 1 ILC subsets and observed that IL-7Rα^+^ ILC1s expressed high levels of CXCR3 (Fig. 3c), raising the possibility that CXCR3 may mediate IL-7Rα^+^ ILC1 recruitment to LNs following hapten sensitization. The mRNA levels of the CXCR3 ligands, *Cxcl9* and *Cxcl10*, increased at 48 h and decreased at 72 h in skin-draining LNs after OXA sensitization (Fig. 3d), consistent with the change in IL-7Rα^+^ ILC1s numbers. As expected, increased recruitment of IL-7Rα^+^ ILC1s to the ILNs and ALNs was abolished in *Cxcr3*^−^^/^^−^ mice (Fig. 3e and Supplementary Fig. 4c), suggesting that migration of IL-7Rα^+^ ILC1s to skin-draining LNs is CXCR3 dependent. To confirm the role of CXCR3 in ILC1-mediated CHS responses, we adoptively transferred liver CXCR3^+^ or CXCR3^−^ group 1 ILC subsets from OXA-sensitized mice into naive recipients that were subsequently challenged with OXA. Only recipients that received CXCR3^+^ cells, rather than the CXCR3^−^ subset, could mediate CHS responses (Supplementary Fig. 4d). Furthermore, CHS responses were reduced in *Cxcr3*^−^^/^^−^ mice (Fig. 3f) and totally abolished in *Rag1*^−^^/^^−^*Cxcr3*^−^^/^^−^ mice (Fig. 3g), demonstrating the critical role of CXCR3 in ILC1-mediated memory responses.

Although liver CD49a^+^ NK cells expressed high levels of CXCR3 (Supplementary Fig. 5a), OXA sensitization did not induce liver CD49a^+^ NK cell recruitment to skin-draining LNs (Supplementary Fig. 5b), suggesting that the increased LN IL-7Rα^+^ ILC1s are not derived from the liver. Splenic cNK cells can be recruited to draining LNs^37^ and cNK cells can convert into ILC1s^38^. To explore whether the increase in LN ILC1s after sensitization resulted from the conversion of cNK cells, naive CD45.2^+^ splenic cNK cells were transferred into naive CD45.1 mice. Upon hapten sensitization, donor cells in recipient LNs did not acquire IL-7Rα and CD49a expression, maintaining their cNK cell phenotype, suggesting that cNK cells did not convert into ILC1s in the CHS model (Supplementary Fig. 5c, d).

Next, we performed parabiotic surgery between CD45.1 and CD45.2 congenic mice to test whether the LN ILC1s were from blood. At 2 weeks after surgery, CD45.2 mice were sensitized with OXA and donor-derived ILC1s increased up to 30% (Supplementary Fig. 5e and 5f). Considering that hapten sensitization can induce a 5–10-fold increase of LN IL-7Rα^+^ ILC1s (Fig. 2c), we conclude that LN ILC1s may be partially recruited from blood.

### IL-7Rα^+^ ILC1s acquire memory potential in skin-draining LNs

**Fig. 4:**
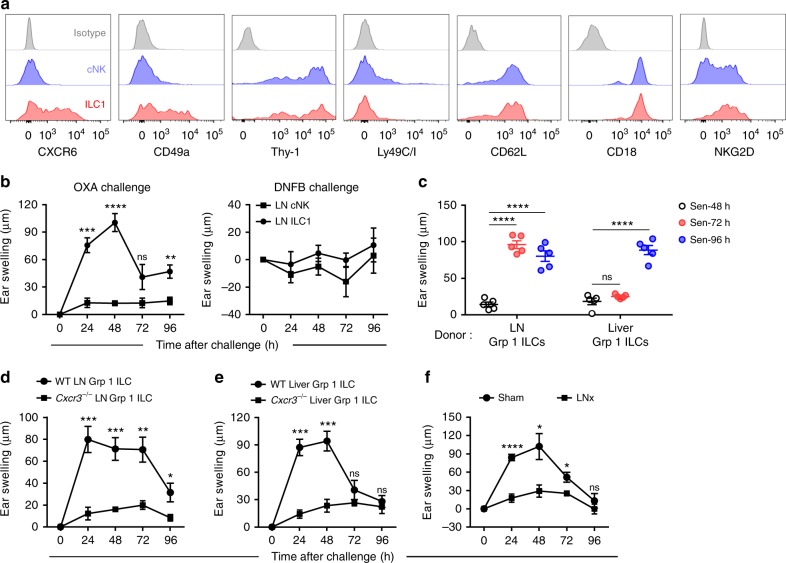
Initial acquisition of immunological memory by IL-7Rα^+^ ILC1s occurs in skin-draining LNs. **a** Expression of CXCR6, CD49a, Thy-1, Ly49C/I, CD62L, CD18, and NKG2D on IL-7Rα^−^ cNK cells and IL-7Rα^+^ ILC1s from ILNs of OXA-sensitized (day 3) WT B6 mice. Data are representative of at least three independent experiments (*n* = 3–5 in each experiment). **b** Ear swelling of WT B6 mice that received 5 × 10^4^ skin-draining LN cNK cells or ILC1s from OXA-sensitized *Rag1*^−^^/^^−^ mice (day 4) and were challenged with OXA or DNFB 1 month later. Data are pooled from two independent experiments (*n* = 4–5 in each group). **c** Ear swelling of WT B6 mice that received 2 × 10^5^ skin-draining LN or liver group 1 ILCs from OXA-sensitized *Rag1*^−^^/^^−^ mice at different time points, followed by challenge with OXA 1 month later. Data are pooled from two independent experiments (*n* = 5 in each group). **d** Ear swelling of WT B6 mice that received 2 × 10^5^ skin-draining LN group 1 ILCs from OXA-sensitized (day 3) WT or *Cxcr3*^−^^/^^−^ mice and were challenged with OXA 1 month later. Data are pooled from two independent experiments (*n* = 4–5 in each group). **e** Ear swelling of WT B6 mice that received 2 × 10^5^ liver group 1 ILCs from OXA-sensitized WT or *Cxcr3*^−^^/^^−^ mice and were challenged with OXA 1 month later. Data are representative of two independent experiments (*n* = 3 in each group). **f** Ear swelling of *Rag1*^−^^/^^−^ mice with surgically removed ILN (LNx) that were sensitized on lower abdomen skin and challenged 4 days later. Data are representative of two independent experiments (*n* = 4 in each group). Means ± SEM are shown. *P < 0.05, **P < 0.01, ***P < 0.001, ****P < 0.0001, two-tailed unpaired Student’s *t* test

The kinetics of ILC1 responses in LNs following hapten sensitization prompted us to investigate whether IL-7Rα^+^ ILC1s initially acquire immunological memory in LNs. Phenotypic analysis revealed that LN IL-7Rα^+^ ILC1s expressed high levels of surface markers associated with group 1 ILC1-mediated CHS responses, including CXCR6, Thy-1, CD62L, CD18, NKG2D, and CD49a; however, LN IL-7Rα^−^ cNK cells lacked the expression of CXCR6 and CD49a (Fig. 4a). Among the Ly49 family, Ly49G2 and Ly49E/F were expressed on LN IL-7Rα^+^ ILC1s at low levels, and the expression of Ly49E and F, of which Ly49E binds to urokinase plasminogen activator^39^ and Ly49F to MHC-I^40^, increased after hapten sensitization (Supplementary Fig. 2d). To further verify whether IL-7Rα^+^ ILC1s in draining LNs can mediate memory responses to haptens, we adoptively transferred IL-7Rα^+^ ILC1s or cNK cells from skin-draining LNs of hapten-sensitized mice into naive recipients, which were challenged with haptens 1 month after transfer. As expected, LN IL-7Rα^+^ ILC1s, rather than IL-7Rα^−^ cNK cells, conferred long-term recall responses in a hapten-specific manner (Fig. 4b).

Next, we sought to determine the chronological order of the acquisition of immunological memory by LN and liver ILC1s. To this end, we adoptively transferred LN or liver group 1 ILCs from *Rag1*^−^^/^^−^ mice that had been sensitized at different time points. We found that, 72 h after hapten sensitization, LN group 1 ILCs already possessed the ability to mediate CHS, while liver group 1 ILCs did not at this time point (Fig. 4c). Robust accumulation of CD45^+^ cells and group 1 ILCs in inflamed skin was consistently observed in mice that received hapten-primed (72 h) LN group 1 ILCs (Supplementary Fig. 6a and 6b). Memory ILC1s emerged in the liver 96 h after hapten sensitization, much later than their emergence in the LNs (Fig. 4c). Furthermore, LN and liver group 1 ILCs from sensitized *Cxcr3*^−^^/^^−^ mice could not mediate hapten-induced skin inflammation, suggesting that blocking recruitment of ILC1s to draining LNs resulted in failure of memory ILC1 generation (Fig. 4d, e). Finally, we found that surgical removal of ILNs led to significantly reduced CHS responses in *Rag1*^−^^/^^−^ mice (Fig. 4f). Overall, these results show that IL-7Rα^+^ ILC1s initially acquire their memory characteristics in skin-draining LNs, rather than in the liver.

### LN-derived memory ILC1s selectively reside in the liver

**Fig. 5:**
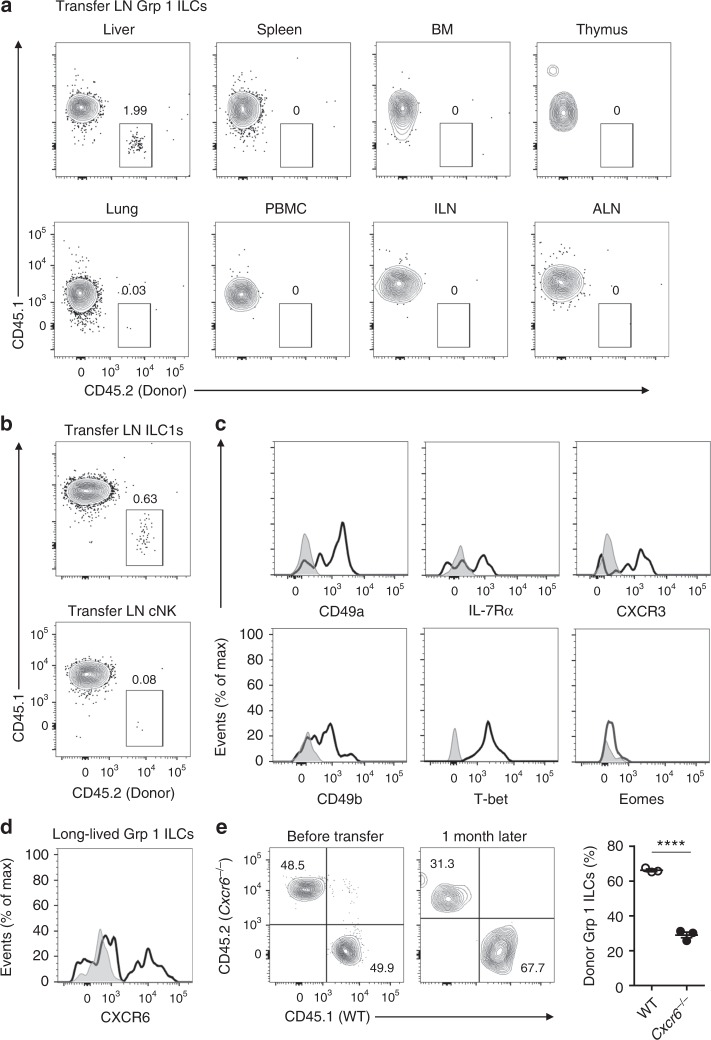
LN memory IL-7Rα^+^ ILC1s selectively reside in the liver. **a** LN group 1 ILCs from OXA-sensitized (72 h) *Rag1*^−^^/^^−^ mice (CD45.2^+^) (2 × 10^5^ cells) were adoptively transferred into CD45.1 mice. Donor cells were analyzed 2 months later. Representative density plots are of cells gated on CD3^−^NK1.1^+^. Data are representative of two independent experiments (*n* = 2 in each experiment). **b** LN IL-7Rα^−^ cNK cells or IL-7Rα^+^ ILC1s from OXA-sensitized (72 h) *Rag1*^−^^/^^−^ mice (CD45.2^+^) (1 × 10^5^ cells) were adoptively transferred into CD45.1 mice. One month later, donor cells were analyzed in recipient livers. Representative density plots are of cells gated on CD3^−^NK1.1^+^. Data are representative of two independent experiments (*n* = 2 in each experiment). **c** Expression of CD127, CD49a, CXCR3, CD49b, T-bet, and Eomes by donor-derived ILC1s in recipient livers of **b**. Histograms are of cells gated on CD3^−^NK1.1^+^CD45.2^+^CD45.1^−^. Data are representative of two independent experiments (*n* = 2 in each experiment). **d** Expression of CXCR6 on donor-derived cells in recipient livers of **a**. Data are representative of two independent experiments (*n* = 2 in each experiment). **e** OXA-sensitized WT (CD45.1^+^) and *Cxcr6*^−^^/^^−^ (CD45.2^+^) LN group1 ILCs (2 × 10^5^ cells) were adoptively transferred into sub-lethally irradiated CD45.1^+^CD45.2^+^ mice; recipient mice were analyzed 1 month later. Representative density plots (left panels) are of liver group 1 ILCs (CD3^−^NK1.1^+^), with host cells (CD45.1^+^CD45.2^+^) excluded. Statistical analysis of results (right panel) showing the percentages of CD45.1^+^ (WT) and CD45.2^+^ (*Cxcr6*^−^^/^^−^) cells among total donor-derived group 1 ILCs (*n* = 3 in each group). Means ± SEM are shown. ****P < 0.0001, two-tailed unpaired Student’s *t* test

As memory ILC1s emerge earlier in skin-draining LNs than in the liver (Fig. 4c) and the LN ILC1 pool is reduced in an apoptosis-independent manner (Fig. 3b), we asked whether hapten-specific ILC1s exited from LNs and migrated to the liver. Sensitized group 1 ILCs from skin-draining LNs (72 h) were adoptively transferred into recipient mice and found to reside only in the recipient livers 2 months later (Fig. 5a), suggesting that the liver serves as a niche for long-term persistence of LN-derived memory ILC1s. We then adoptively transferred equal numbers of sensitized (72 h) IL-7Rα^+^ ILC1s and IL-7Rα^−^ cNK cells from skin-draining LNs into recipient mice. The frequency of LN-derived ILC1s in recipient livers was significantly higher than that of LN-derived cNK cells (Fig. 5b), with ILC1s identified by their expression of IL-7Rα and T-bet and lack of Eomes (Fig. 5c). These data suggest that LN IL-7Rα^+^ ILC1s represent a long-lived subset among group 1 ILCs and that the liver serves as a niche for their long-term survival.

Notably, a substantial proportion of LN ILC1s and most liver ILC1s constantly expressed CXCR6, while cNK cells lacked CXCR6, even after hapten sensitization (Figs. 4a, 5d, Supplementary Fig. 7a and 7b). The CXCR6 ligand, CXCL16, is constitutively expressed in the liver^41^. Comparison of the migration of *Cxcr6*^−^^/^^−^ LN group 1 ILCs with their wild-type (WT) counterparts showed that migration of *Cxcr6*^−^^/^^−^ LN ILC1s to the recipient liver was inhibited by approximately 30% (Fig. 5e), suggesting that the residency of LN-derived memory ILC1s in the liver is partly dependent on CXCR6.

**Fig. 6:**
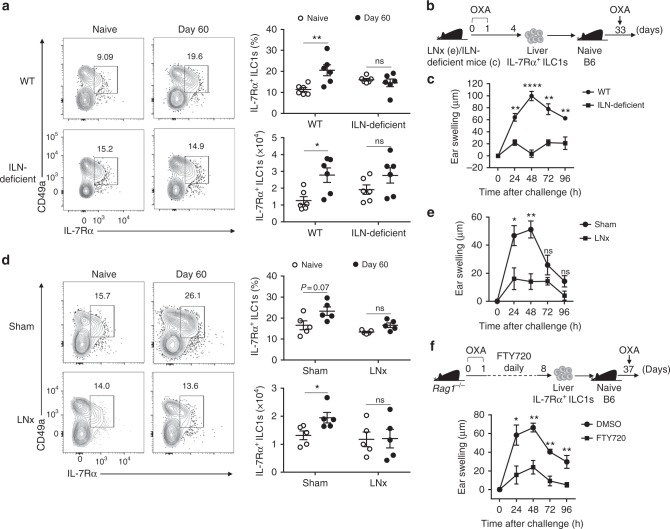
Liver IL-7Rα^+^ ILC1-mediated memory responses are abolished in the absence of draining LNs. **a** ILN-deficient mice were sensitized with OXA and analyzed 2 months later. Representative dot plots (left panels) showing CD49a and IL-7Rα expression on liver CD3^−^NK1.1^+^NKp46^+^ cells. Statistical analysis of results (right panels) showing the percentages and absolute numbers of liver IL-7Rα^+^ ILC1s. Data are pooled from two independent experiments (*n* = 6 in each group). **b** Schematic of experiments for **c**, **e**. **c** Ear swelling of WT B6 mice that received 5 × 10^4^ liver IL-7Rα^+^ ILC1s from OXA-sensitized (96 h) ILN-deficient mice and were challenged 1 month later. Data are pooled from two independent experiments (*n* = 4 in each group). **d** WT B6 mice underwent surgical removal of ILNs (LNx) or sham operations and were subsequently sensitized with OXA. Two months later, the percentages and absolute numbers of liver IL-7Rα^+^ ILC1s were analyzed. Dot plots are of cells gated on CD3^−^NK1.1^+^. Data are pooled from two experiments (*n* = 5 in each group). **e** CHS of naive B6 mice that received 5 × 10^4^ liver IL-7Rα^+^ ILC1s from OXA-sensitized (96 h) LNx *Rag1*^−^^/^^−^ mice and were challenged with OXA 1 month later. Data are pooled from two experiments (*n* = 6 in each group). **f** *Rag1*^−^^/^^−^ mice were sensitized (days 0, 1) with OXA and i.p. injected with FTY720 daily for 6 consecutive days (days 2–7). On day 8, 2–3 × 10^4^ liver IL-7Rα^+^ ILC1s from FTY720-treated *Rag1*^−^^/^^−^ mice were adoptively transferred into naive B6 mice; recipient mice were then challenged with OXA and ear swelling was measured. Data are pooled from two independent experiments (*n* = 3–6 in each group). Means ± SEM are shown. *P < 0.05, **P < 0.01, ****P < 0.0001, two-tailed unpaired Student’s *t* test

To determine whether liver memory IL-7Rα^+^ ILC1s are derived from skin-draining LNs, we analyzed liver IL-7Rα^+^ ILC1 responses to haptens in the absence of skin-draining LNs. We generated ILN-deficient mice by injecting LTβR-Ig into pregnant B6 mice. The hapten-induced increase of liver memory IL-7Rα^+^ ILC1s was abrogated in ILN-deficient mice (Fig. 6a), and liver IL-7Rα^+^ ILC1s from sensitized (96 h) ILN-deficient mice failed to confer hapten-induced memory responses (Fig. 6b, c). Next, we performed lymphadenectomy of ILN. No increase of IL-7Rα^+^ ILC1s was observed in the livers of mice with ILNs removed following OXA sensitization (Fig. 6d). Furthermore, liver IL-7Rα^+^ ILC1s from OXA-sensitized (96 h) mice with ILNs removed could not transfer CHS responses to naive mice (Fig. 6b, e). The chemical, FTY720, blocks lymphocyte egress from LNs by inhibiting sphingosine-1-phosphate receptor 1 (S1PR1), leading to retention of lymphocytes in LNs^42^. We found that IL-7Rα^+^ ILC1s were increased in the LNs of FTY720-treated mice (Supplementary Fig. 8a, b), suggesting that IL-7Rα^+^ ILC1 egress from LNs relies on S1PR1, similar to T cells. Moreover, adoptive transfer experiments revealed that liver IL-7Rα^+^ ILC1s from FTY720-treated mice could only induce weak skin inflammation compared to those from control mice (Fig. 6f). Collectively, these results indicate that the liver represents a unique niche for the residency and long-term homeostasis of LN-derived memory ILC1s.

### IL-7R signaling is necessary for memory ILC1 longevity

**Fig. 7:**
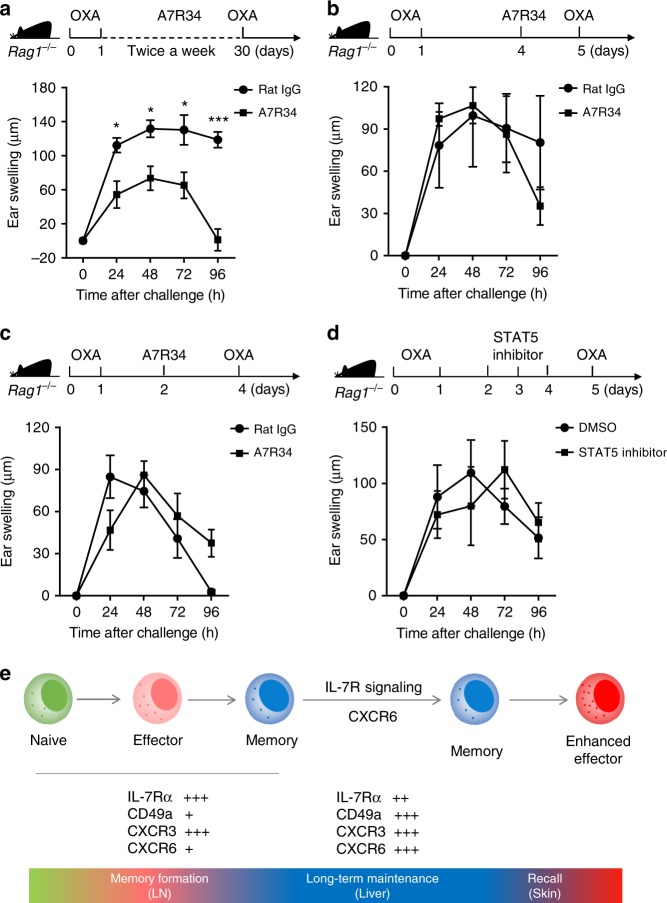
IL-7R signaling is required for long-term maintenance of memory ILC1s. **a** Ear swelling of *Rag1*^−^^/^^−^ mice that were i.p. injected with A7R34 antibody twice a week for 4 weeks, followed by OXA challenge (*n* = 5 in each group). **b** Ear swelling of *Rag1*^−^^/^^−^ mice i.p. injected with A7R34 antibody 24 h before OXA challenge (*n* = 3–4 in each group). **c** Ear swelling of *Rag1*^−^^/^^−^ mice i.p. injected with A7R34 antibody on day 2 and challenged on day 4 after sensitization (*n* = 4 in each group). **d** Ear swelling of *Rag1*^−^^/^^−^ mice that were i.p. injected with STAT5 inhibitor daily since day 2 and challenged on day 5 after sensitization (*n* = 4 in each group). **e** Summary of the generation and maintenance of memory ILC1s. Means ± SEM are shown. *P < 0.05, ***P < 0.001, two-tailed unpaired Student’s *t* test

IL-7R signaling is dispensable for ILC1 development^27–29^. Given the critical role of IL-7R signaling in the long-term maintenance of memory T cells^30,31^, we investigated its contribution to the longevity of memory ILC1s. OXA-sensitized *Rag1*^−^^/^^−^ mice were continuously injected with anti-IL-7Rα antibody (A7R34) for 4 weeks, followed by OXA challenge. Interestingly, long-term blockade of IL-7Rα reduced skin inflammation (Fig. 7a and Supplementary Fig. 8c), suggesting that the longevity of memory ILC1s does rely on IL-7R signaling. As liver sinusoidal endothelial cells (LSECs) constitutively secrete IL-7^43^, liver sinusoids may provide a niche for the long-term maintenance of memory IL-7Rα^+^ ILC1s. Blocking IL-7Rα in OXA-sensitized *Rag1*^−^^/^^−^ mice 24 h before OXA challenge did not affect CHS responses, suggesting that IL-7R signaling is not required for the secondary responses of memory ILC1s (Fig. 7b). Next, we injected *Rag1*^−^^/^^−^ mice with an anti-IL-7Rα blocking antibody, 2 days after hapten sensitization, and challenged mice on day 4; however, CHS responses did not differ between these mice and controls, indicating that blockade of IL-7Rα had no effect on memory ILC1 formation (Fig. 7c). IL-7R mainly activates the transcription factor, signal transducer and activator of transcription factor 5 (STAT5)^26;^ however, inhibition of STAT5 during the sensitization phase did not affect ILC1-mediated CHS responses (Fig. 7d). Overall, these data reveal a critical role for IL-7R signaling in the long-term maintenance of memory ILC1s (Fig. 7e).

## Discussion

In this study, we present a road map for the formation and maintenance of liver memory ILC1s. Upon hapten application to the skin, skin-resident DCs immediately take up haptens, or hapten-self protein complexes, and migrate to draining LNs within 24 h^44^. Activated DCs or stromal cells may provide a source of CXCL10 and CXCL9^45^. Our studies demonstrate that hapten sensitization induces rapid IL-7Rα^+^ ILC1 recruitment to skin-draining LNs in a CXCR3-dependent manner. In skin-draining LNs, IL-7Rα^+^ ILC1s are primed and acquire immunological memory, then the memory IL-7Rα^+^ ILC1s exit draining LNs via S1PR1 and selectively reside in the hepatic sinusoids via CXCR6–CXCL16 interaction. Memory ILC1s maintain longevity in the liver through IL-7R signaling, which allows the long-term residency of memory ILC1s in the liver. Once the skin encounters the same hapten, memory IL-7Rα^+^ ILC1s accumulate at effector sites and mediate robust allergic skin inflammation.

Our present study shows that IL-7Rα^+^ ILC1 recruitment and priming in the LNs require CXCR3, and in *Cxcr3*^−^^/^^−^ mice or ILN-deficient mice, liver ILC1s failed to mediate CHS responses; however, we cannot exclude the possibility that naive liver ILC1s can respond to haptens and acquire memory, relying on LNs and CXCR3. Indeed, previous studies have reported that other cell types, such as plasmacytoid DC precursors, migrate to draining LNs in a CXCR3-dependent manner^46^ and that DC-derived signals are required to prime NK cells in LNs^47,48^. These factors may also have impacted the reduced CHS responses in *Cxcr3*^−^^/^^−^ mice. Further investigations are needed to confirm whether two parallel systems of liver- and LN-derived memory ILC1s exist.

Memory ILC1s and central memory T (T_CM_) cells exhibit both similarities and differences in their migratory properties. Comparison of the number and functional status of ILC1s from various anatomical sites revealed that ILC1s are initially increased and activated in draining LNs after sensitization, suggesting that they are primed in LNs, in a process analogous to T cell priming. Interestingly, both memory ILC1s and T_CM_ cells exhibit tissue migration and residency preferences, despite their different destinations. BM is the preferential homing site for T_CM_ cells^49^, whereas memory ILC1s migrate along a LN–liver axis. These different homing sites may be attributable to differential expression of homing receptors and the tissue distribution of their corresponding ligands. Indeed, our findings reveal that residency of LN-derived memory ILC1s in the liver partly relies on CXCR6, a chemokine receptor of CXCL16, which is highly expressed in the liver. Of note, recent studies have revealed a critical role for tissue-resident memory T (T_RM_) cells, which can persist in the skin after virus clearance^50^. As the skin contains abundant CD49a^+^ tissue-resident NK (trNK) cells under steady-state conditions^34^, it will be of interest to investigate whether trNK cells can acquire immunological memory locally, in a manner similar to T_RM_ cells.

Previous studies have demonstrated that Ly49H^+^ cNK cells can mediate memory responses to MCMV infection^8^. The dynamics of MCMV-specific Ly49H^+^ NK cells are particularly comparable to those of antigen-specific CD8^+^ T cells. Following MCMV infection, Ly49H^+^ NK cells proliferate dramatically, expanding to a population peak on day 7, and then undergo a contraction phase, resulting in a minor population surviving and being maintained as memory cells^8,9^. In contrast, haptens induce the greatest increase of LN ILC1s on day 2 after sensitization, and these cells acquire immunological memory as early as day 3. Thus the process of formation of memory ILC1s occurs more rapidly than those generating MCMV-specific memory NK cells and memory CD8^+^ T cells.

Although hapten application to the skin induces increased numbers of ILC1s in draining LNs, the proliferation rate of this population remains unchanged. As NK cells can migrate to DC-draining LNs^22^, we hypothesized that ILC1s may act similarly after hapten sensitization. As expected, we found that migration of ILC1s to draining LNs was dependent on CXCR3. There are two possible means of ILC1 entrance into LNs: afferent lymphatic vessels and high endothelial venules (HEV). Peripheral CCR7^+^ T cells egress into LNs via HEV, while infected tissue-derived DCs egress into LNs via afferent lymphatic vessels. Using parabiotic mice, we demonstrated that LN IL-7Rα^+^ ILC1 recruitment could, in part, be from blood. A previous study using mice expressing photo-convertible proteins demonstrated that γδT17 cells can migrate from skin to skin-draining LNs in an imiquimod-induced skin inflammation model^51^. It will be of interest to determine whether skin-resident IL-7Rα^+^ ILC1s can be recruited to skin-draining LNs following sensitization.

Although IL-7R is not required for steady-state ILC1 development^27–29^, our study reveals that IL-7Rα is a marker of memory ILC1s that is critical for their long-term homeostasis. Defects in IL-7Rα do not influence the expression of BCL-2 in ILC1s^29^; therefore, we assume that a BCL-2-independent mechanism may exist for IL-7Rα-mediated long-term homeostasis of memory ILC1s. IL-7R signaling may induce TAG synthesis to promote FAO metabolism in memory ILC1s, similar to memory CD8^+^ T cells^32^. LSECs and hepatocytes can secrete large amounts of IL-7^43,52,53^. Thus the enrichment for IL-7 alongside CXCL16 expression in the liver provides a suitable environment for the residency and long-term maintenance of LN-derived memory IL-7Rα^+^ ILC1s.

Murine CHS is a well-established and useful model for the study of ACD, one of the most common human skin diseases. In patients with ACD, IFN-γ- and TNF-producing CD3^−^CD56^high^CD16^−^ cells accumulate in the inflamed skin^18^. Although it is unclear whether these cells are ILC1s or cNK cells, they exhibit high levels of CXCR3 expression^18^, which is an important marker of murine memory IL-7Rα^+^ ILC1s. Moreover, IL-7Rα is primarily expressed on CD56^high^, but not CD56^dim^, NK cells^19^; thus it is possible that human ILC1s may contribute to allergic skin inflammation. Regardless, our findings provide new insights into the process of memory ILC1 formation and maintenance and offer a foundation for further investigation of the role of ILC1s in human disease.

## Methods

### Mice

C57BL/6 mice (Stock No. 000664) were purchased from the Shanghai Laboratory Animal Center (SLAC, Chinese Academy of Sciences). *Rag1*^−^^/^^−^ mice were obtained from the Model Animal Research Center (Nanjing University). *CD45.1* mice (Stock No. 002014) and *Cxcr6*^gfp/gfp^ mice (Stock No. 005693) were purchased from the Jackson Laboratory. *Cxcr3*^−^^*/*^^−^ mice were kindly provided by Professor Zhexiong Lian (University of Science and Technology of China). *Rag1*^−^^/^^−^*Cxcr3*^−^^/^^−^ and *CD45.1*^+^*CD45.2*^+^ mice were bred in house on a C57BL/6 background. For most experiments, 3–10 age- and sex-matched mice were used. No exclusion, randomization, or blinding were performed in this study. We maintained mice in a specific pathogen-free facility, according to the guidelines for experimental animals. The Ethics Committee at the University of Science & Technology of China approved all experiments.

### Antibody staining and flow cytometry

Antibody information is summarized in Supplementary Table 1. Prior to staining with antibodies, cells were incubated with rat serum for 30 min to block Fc receptors. For intracellular staining, the FOXP3 Staining Kit (eBioscience) was used. Flow cytometry was performed on an LSR II (BD Biosciences) or Sony SP6800 Spectral Analyzer (Supplementary Fig 6). Data were analyzed using the FlowJo software (Tree Star).

### Cell isolation

Liver tissues were passed through a 200-gauge stainless steel mesh. Cells were then resuspended in 40% Percoll (GE Healthcare), gently overlaid onto 70% Percoll, and collected at the interface layer after centrifugation. Liver mononuclear cells were collected from the interphase. For LN cell isolation, LNs were teased using fine forceps and mechanically disrupted. LN cells were digested for 25 min at 37 °C in RPMI with 500 μg/ml Collagenase IV (Sigma) and 25 μg/ml DNase I (Sigma). Splenocytes were obtained by passing spleens through stainless steel mesh and lysing erythrocytes. BM cells were obtained by flushing femurs and lysing erythrocytes. Peripheral blood mononuclear cells were obtained by lysing the erythrocytes in blood samples.

### Cell sorting and transfer

A FACS Aria (BD Biosciences) was used to sort IL-7Rα^+^ ILC1s, IL-7Rα^−^ LrNK cells, or cNK cells. The purity of sorted cell populations was ≥95%, as verified by flow cytometry. ILC1s, LrNK cells, cNK cells, or total group1 ILCs were adoptively transferred into sub-lethally irradiated (6.5 Gy) recipient mice, followed by induction of CHS responses and analysis of the donor cell phenotype.

### CHS

On days 0 and 1, mice were sensitized by painting shaved abdomen skin with 50 μl 5% OXA (Sigma) in a solution of acetone/methanol (1:1), 50 μl 0.5% DNFB (Solarbio) in acetone, or 100 μl 0.5% FITC (Sigma) in a solution of acetone/dibutyl phthalate (1:1). Later (48 h, 72 h, 96 h, or 2 months), these mice were used as donors for cell sorting and transfer or, alternatively, for analysis of group 1 ILCs from multiple tissues. Recipient mice were challenged by painting the skin of their right ears with 20 μl 0.2% DNFB or 20 μl 1% OXA; left ears were painted with solvent as controls. Ear thickness was measured every 24 h using a micrometer. To evaluate acute hapten-induced irritation, background swelling was measured in naive mice treated with phosphate-buffered saline. Hapten-specific ear swelling was calculated as follows: (treated ear thickness − control ear thickness) − background swelling.

### Real-time PCR

Total RNA was extracted using TRIzol reagent (Invitrogen). cDNA synthesis was performed using Moloney Murine Leukemia Virus Reverse Transcriptase (Invitrogen) and random primers, according to the manufacturer’s instructions. Quantitative real-time PCR was performed on a Light Cycler (Roche Diagnostics) using SYBR Premix Ex Taq (TaKaRa). The primer pairs used were as follows: CXCL9 forward, 5′-CAAATCCCTCAAAGACCTCAAAC-3′; CXCL9 reverse, 5′-GATCTCCGTTCTTCAGTGTAGC-3′; CXCL10 forward, 5′-TCATCCCTGCGAGCCTAT-3′; and CXCL10 reverse, 5′-CTTGATGGTCTTAGATTCCGGAT-3′.

### In vivo treatment

FTY720 (Sigma) was used to block S1PR1. WT or *Rag1*^−^^/^^−^ mice were injected intraperitoneally (i.p.) with 20 μg FTY720 every 24 h for 6 days. *Rag1*^−^^/^^−^ mice were injected i.p. with 100 μg STAT5 inhibitor (Santa Cruz Biotechnology) every 24 h for 3 days to inhibit STAT5 signaling. To block IL-7Rα signaling, *Rag1*^−^^/^^−^ mice were injected i.p. with 200 μg IL-7Rα blockade antibody (A7R34, Bio X Cell), using single or continuous injections, as indicated.

### Parabiosis

CD45.1 and CD45.2 male mice of the same age were parabiosed. Both mice were anesthetized and a longitudinal incision was made along the lateral aspect of each mouse. Mice were then joined at the elbow and knee, and the incision was closed with wound clips. Mice were allowed to recover for 2 weeks before hapten administration.

### Generation of ILN-deficient mice

LTβR-Ig was a gift from Professor Yangxin Fu (UT Southwestern Medical Center, Dallas, TX, USA). Pregnant B6 mice were injected with 100 μg LTβR-Ig intravenously at gestational day 13.5, which completely prevented the formation of ILNs in newborn mice^54,55^.

### Statistics

All data are presented as means ± SEM. The statistical significance of differences was determined by two-tailed unpaired Student’s *t* tests for two groups and one-way analysis of variance for three groups (GraphPad). *P* values <0.05 were considered significant.

## Electronic supplementary material

Supplementary Information

## Data Availability

All data that support the findings of this study are available from the corresponding author on reasonable request.
