# Author Correction: Memory formation and long-term maintenance of IL-7Rα^+^ ILC1s via a lymph node-liver axis

**DOI:** 10.1038/s41467-018-07854-y

**Published:** 2019-01-08

**Authors:** Xianwei Wang, Hui Peng, Jingjing Cong, Xuefu Wang, Zhexiong Lian, Haiming Wei, Rui Sun, Zhigang Tian

**Affiliations:** 1 Division of Molecular Medicine, Hefei National Laboratory for Physical Sciences at Microscale, The CAS Key Laboratory of Innate Immunity and Chronic Disease, School of Life Sciences, University of Science and Technology of China, Hefei, 230027 Anhui China; 2 Institue of Immunology, University of Science and Technology of China, Hefei, 230027 Anhui China

Correction to: *Nature Communications* (2018) 9, 4854; 10.1038/s41467-018-07405-5, published online 19 November 2018

The original version of this Article contained errors in Figs. 1 and 6. In Fig. 1c, the label ‘CD49a^+^IL-7Rα^+^ ILC1’ incorrectly read ‘CD49a^+^IL-7Rα^−^ ILC1’. In Fig. 6b, the label ‘LNx (e)/ILN-deficient mice (c)’ incorrectly read ‘LNx (c)/ILN-deficient mice (e)’. These errors have now been corrected in both the PDF and HTML versions of the Article.

