# Supplementary material for: Memory formation and long-term maintenance of IL-7Rα+ ILC1s via a lymph node-liver axis
Source: Nat Commun. 2018 Nov 19;9:4854. doi: 10.1038/s41467-018-07405-5 (PMC6242895; doi:10.1038/s41467-018-07405-5)
Supplement: Supplementary file 1 — Supplementary Information [file 41467_2018_7405_MOESM1_ESM.pdf]

Supplementary Information

Memory formation and long-term maintenance of IL-7Rα$^+$ ILC1s via a lymph node-liver axis

Xianwei Wang[1,2], Hui Peng[1,2*], Jingjing Cong[1,2], Xuefu Wang[1,2], Zhexiong Lian[1,2], Haiming Wei[1,2], Rui Sun[1,2*], and Zhigang Tian[1,2*]

[1]Division of Molecular Medicine, Hefei National Laboratory for Physical Sciences at Microscale, the CAS Key Laboratory of Innate Immunity and Chronic Disease, School of Life Sciences, University of Science and Technology of China, Hefei, Anhui, China

[2]Institue of Immunology, University of Science and Technology of China, Hefei, Anhui, China

[*]Correspondence: huipeng@mail.ustc.edu.cn (H.P.), sunr@ustc.edu.cn (R.S.), tzg@ustc.edu.cn (Z.T.)

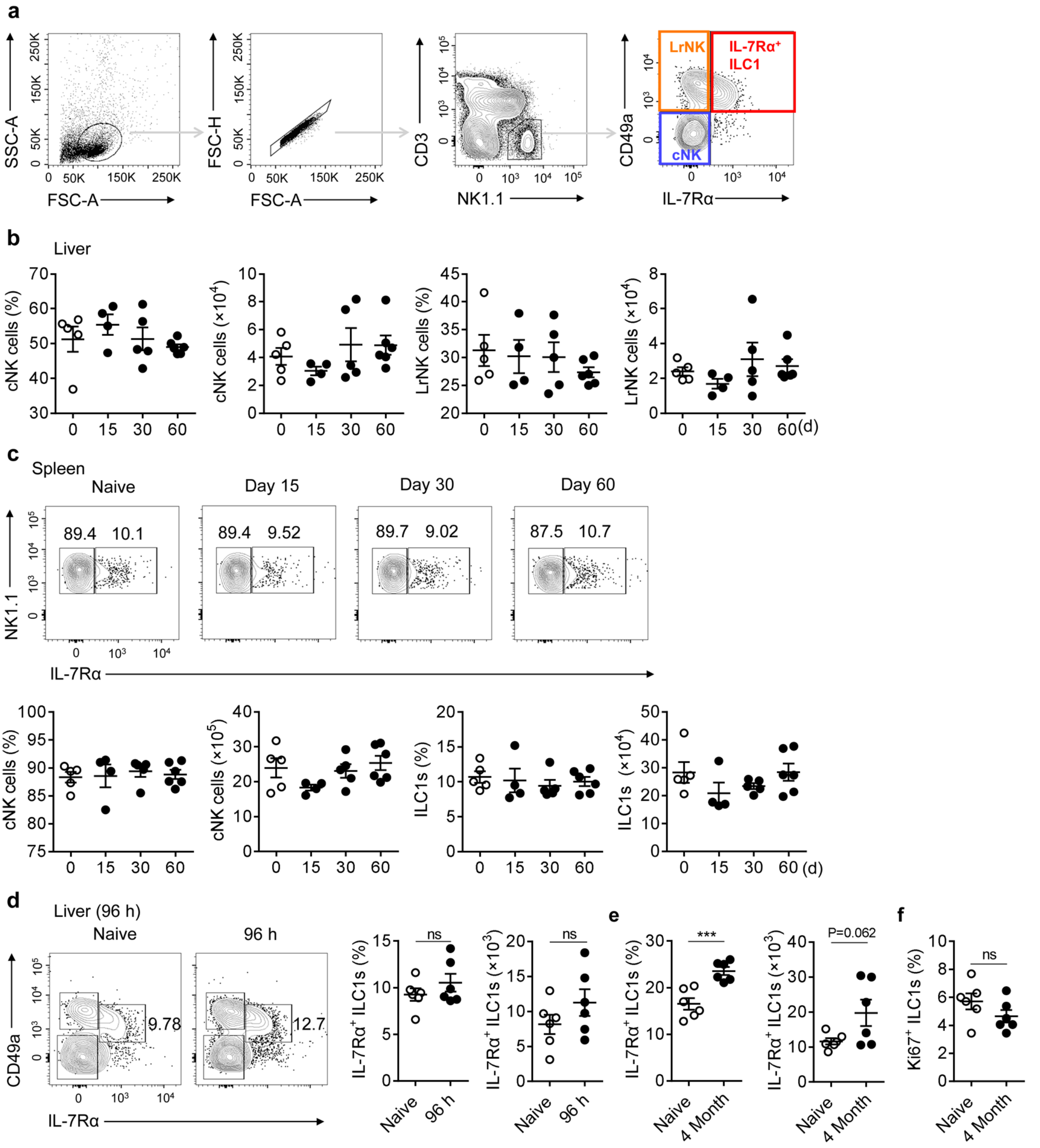

Supplementary Figure 1. Kinetics of liver and spleen group 1 ILC subsets after OXA sensitization.

(**a**) FACS gating/sorting strategies for liver group 1 ILC subsets. (**b**) The percentages and absolute numbers of liver cNK cells ($CD3^{-}NK1.1^{+}NKp46^{+}CD49a^{-}IL\text{-}7R\alpha^{-}$) and IL-7Rα^{-} LrNK cells ($CD3^{-}NK1.1^{+}NKp46^{+}CD49a^{+}IL\text{-}7R\alpha^{-}$) were assessed after OXA sensitization in WT B6 mice. Data are

representative of two independent experiments (n = 4–6 in each group). (**c**) Representative density plots (top panels) showing CD49a and IL-7Rα expression on splenic $CD3^{-}NK1.1^{+}NKp46^{+}$ cells after OXA sensitization in WT B6 mice. Statistical analysis of results (bottom panels) showing the percentages and absolute numbers of splenic cNK cells ($CD3^{-}NK1.1^{+}NKp46^{+}IL\text{-}7R\alpha^{-}$) and ILC1s ($CD3^{-}NK1.1^{+}NKp46^{+}IL\text{-}7R\alpha^{+}$). Data are representative of two independent experiments (n = 4–6 in each group). (**d**) The percentages and absolute numbers of liver IL-7Rα^{+} ILC1s 96 h after OXA sensitization. Representative dot plots showing CD49a and IL-7Rα expression on liver $CD3^{-}NK1.1^{+}$ cells at different time points in B6 mice. Data are pooled from two independent experiments (n = 6 in each group). (**e**) The percentages and absolute numbers of liver IL-7Rα^{+} ILC1s in B6 mice 4 months after sensitization. Data are pooled from two independent experiments (n = 6 in each group). (**f**) Percentages of Ki67^{+} liver ILC1s in B6 mice 4 months after sensitization. Data are pooled from two independent experiments (n = 6 in each group). Means ± SEM are shown. ***$P < 0.001$, two-tailed unpaired Student's *t*-test.

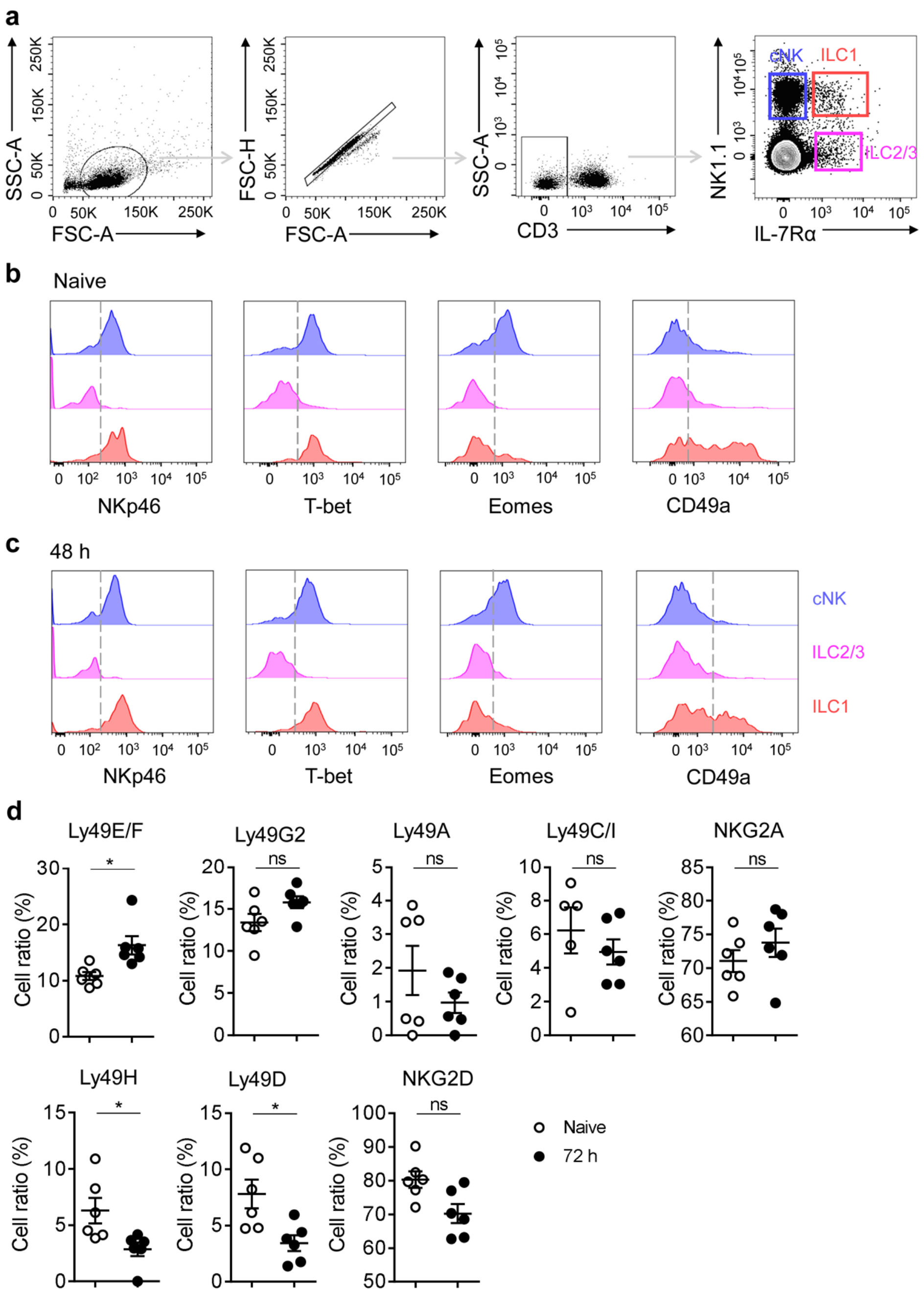

Supplementary Figure 2. Analysis of LN ILC characteristics.

(**a**) FACS gating/sorting strategies for LN group 1 ILC subsets. (**b, c**) Expression of the indicated molecules on LN ILC subsets from naïve (**b**) or OXA-sensitized (**c**) mice. Data are representative of at least three experiments (n = 3–5 in each experiment). (**d**) Percentages of Ly49 or NKG2

receptor positive IL-7Rα^{+} ILC1s from ILNs of naïve and OXA-sensitized (day 3) WT B6 mice (n = 3 in each group). Means ± SEM are shown. *P < 0.05, two-tailed unpaired Student's *t*-test.

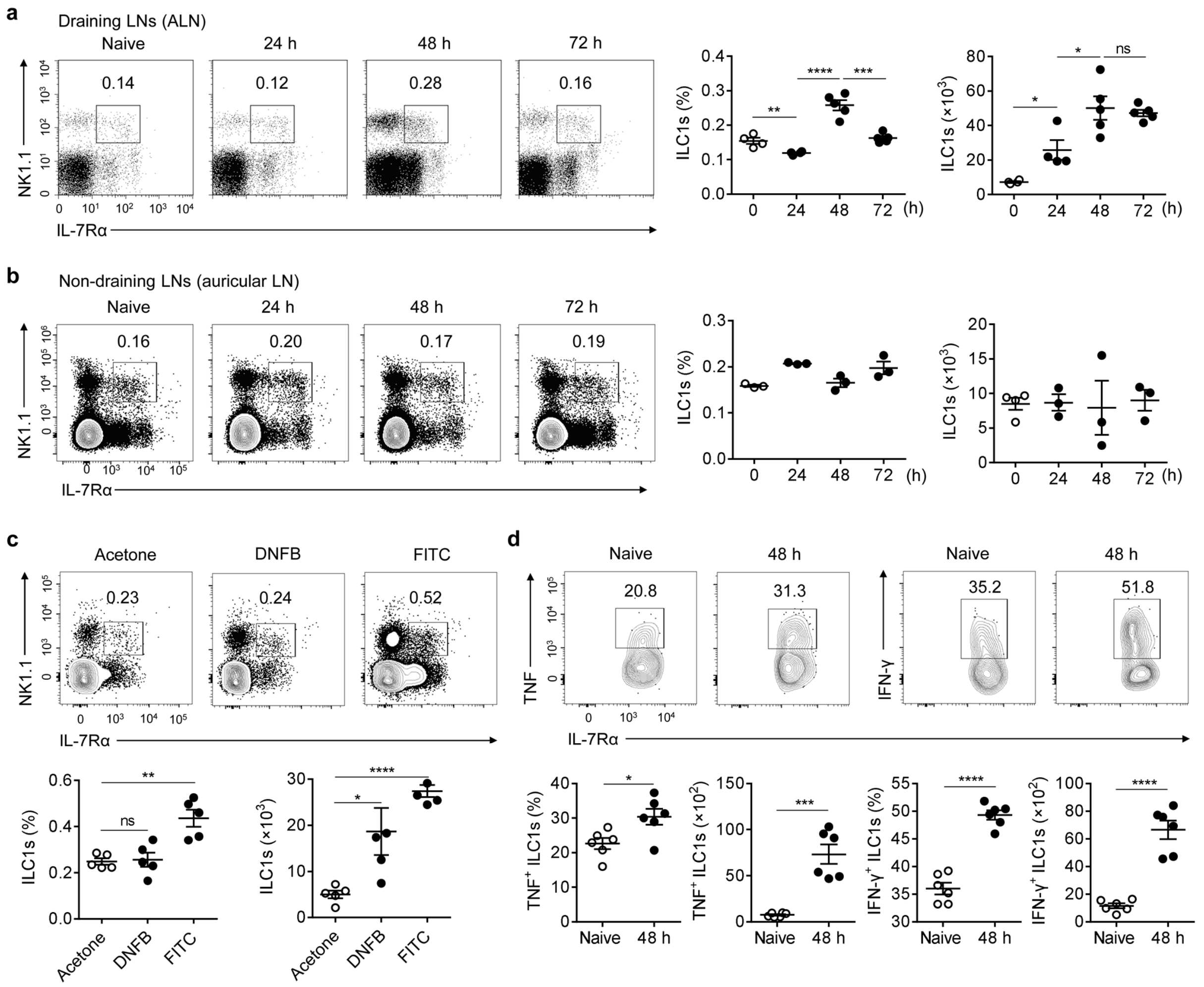

Supplementary Figure 3. Haptens induce IL-7Rα$^+$ ILC1 priming in ALNs.

(**a**) Representative dot plots (left panels) showing NK1.1 and IL-7Rα expression on ALN CD3$^-$ cells from OXA-sensitized WT B6 mice. Numbers in dot plots represent the percentages of ILN IL-7Rα$^+$ ILC1s cells among CD45$^+$ cells. Statistical analysis of results (right panels) showing the percentages and absolute numbers of ALN IL-7Rα$^+$ ILC1s. Data are representative of at least three independent experiments (n = 3–5 in each group). (**b**) Representative density plots (left panels) showing NK1.1 and IL-7Rα expression on auricular LN CD3$^-$ cells from OXA-sensitized WT B6 mice. Numbers in dot plots represent the percentages of ILN IL-7Rα$^+$ ILC1s cells among CD45$^+$ cells. Statistical analysis of results (right panels) showing the percentages and absolute numbers of auricular LN IL-7Rα$^+$ ILC1s. Dot plots are of cells gated on CD3$^-$ (n = 3–4 in each group). (**c**) Representative density plots (top panels) showing NK1.1 and IL-7Rα expression on ALN CD3$^-$

cells from DNFB or FITC sensitized WT B6 mice. Numbers in dot plots represent the percentages of ILN IL-7Rα$^+$ ILC1s cells among CD45$^+$ cells. Statistical analysis of results (bottom panels) showing the percentages and absolute numbers of ALN IL-7Rα$^+$ ILC1s. Data are representative of two experiments (n = 4–5 in each group). (**d**) Representative density plots (top panels) showing expression of IFNγ and TNF by ALN IL-7Rα$^+$ ILC1s from naïve or OXA sensitized (48 h) mice after stimulation of ALN cells with PMA/Ion (for TNF detection) or IL-12/IL-18 (for IFNγ detection). Statistical analysis of results (bottom panels) showing the percentages and absolute numbers of TNF-producing or IFNγ-producing ALN IL-7Rα$^+$ ILC1s. Data are representative of two experiments (n = 4–5 in each group). Means ± SEM are shown. *$P < 0.05$, **$P < 0.01$, ***$P < 0.001$, ****$P < 0.0001$, two-tailed unpaired Student's *t*-test.

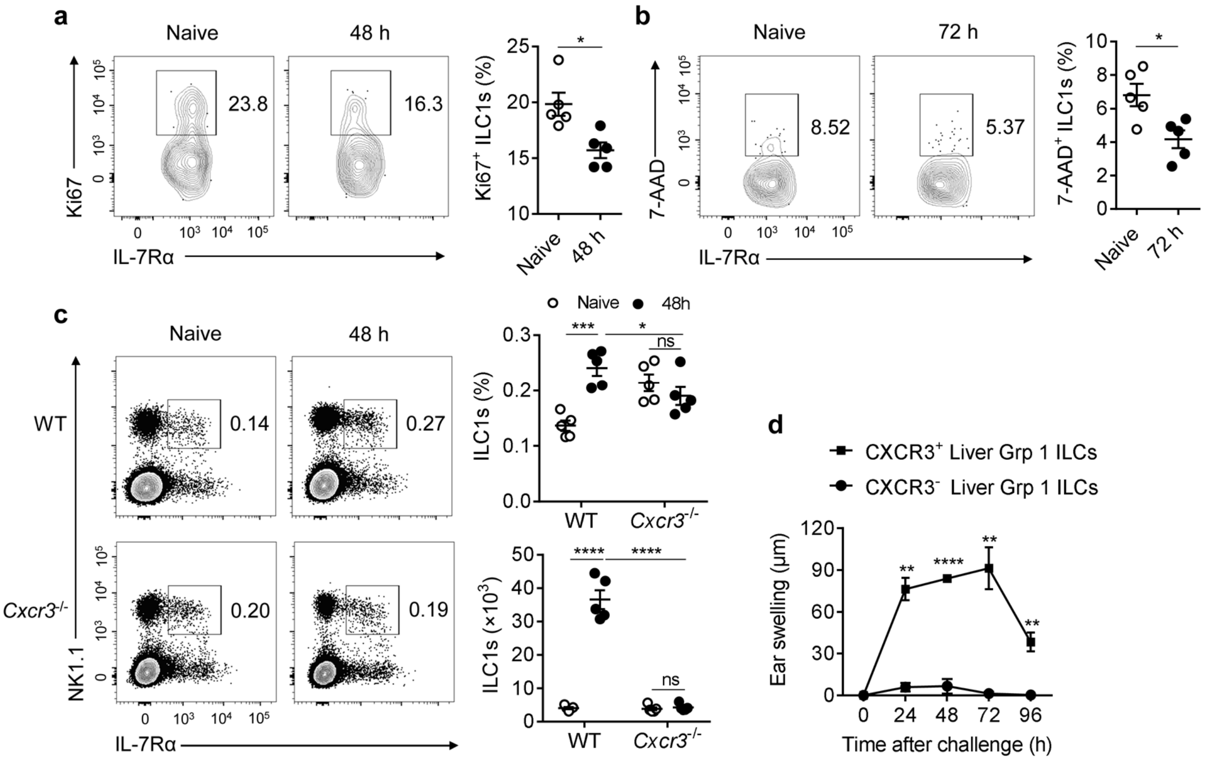

Supplementary Figure 4. Critical role for CXCR3 in IL-7Rα$^+$ ILC1-mediated CHS responses

(**a, b**) Representative density plots (left panels) showing Ki67 (A) or 7-AAD (B) staining on ALN CD3$^-$NK1.1$^+$IL-7Rα$^+$ cells from OXA-sensitized (**a**: 48 h; **b**: 72 h) WT B6 mice. Statistical analysis of results (right panels) showing percentages of Ki67$^+$ (**a**) or 7-AAD$^+$ (**b**) cells among ALN ILC1s. Data are representative of two independent experiments (n = 4–5 in each group). (**c**) Representative density plots (left panels) showing CD49a and IL-7Rα expression on ALN CD3$^-$ cells from OXA-sensitized (48 h) WT or *Cxcr3*$^{-/-}$ mice. Numbers in dot plots represent the percentages of ALN IL-7Rα$^+$ ILC1s among CD45$^+$ cells. Statistical results (right panels) showing the percentages and absolute numbers of ALN IL-7Rα$^+$ ILC1s. Data are representative of two experiments (n = 4–5 in each group). (**d**) Ear swelling of WT B6 mice that received 1 × 10^5 liver CXCR3$^+$ or CXCR3$^-$ group 1 ILCs from OXA-sensitized *Rag1*$^{-/-}$ mice. Data are pooled from two experiments (n = 3 in each group). *$P < 0.05$, **$P < 0.01$, ***$P < 0.001$, and ****$P < 0.0001$. Means ± SEM are shown.

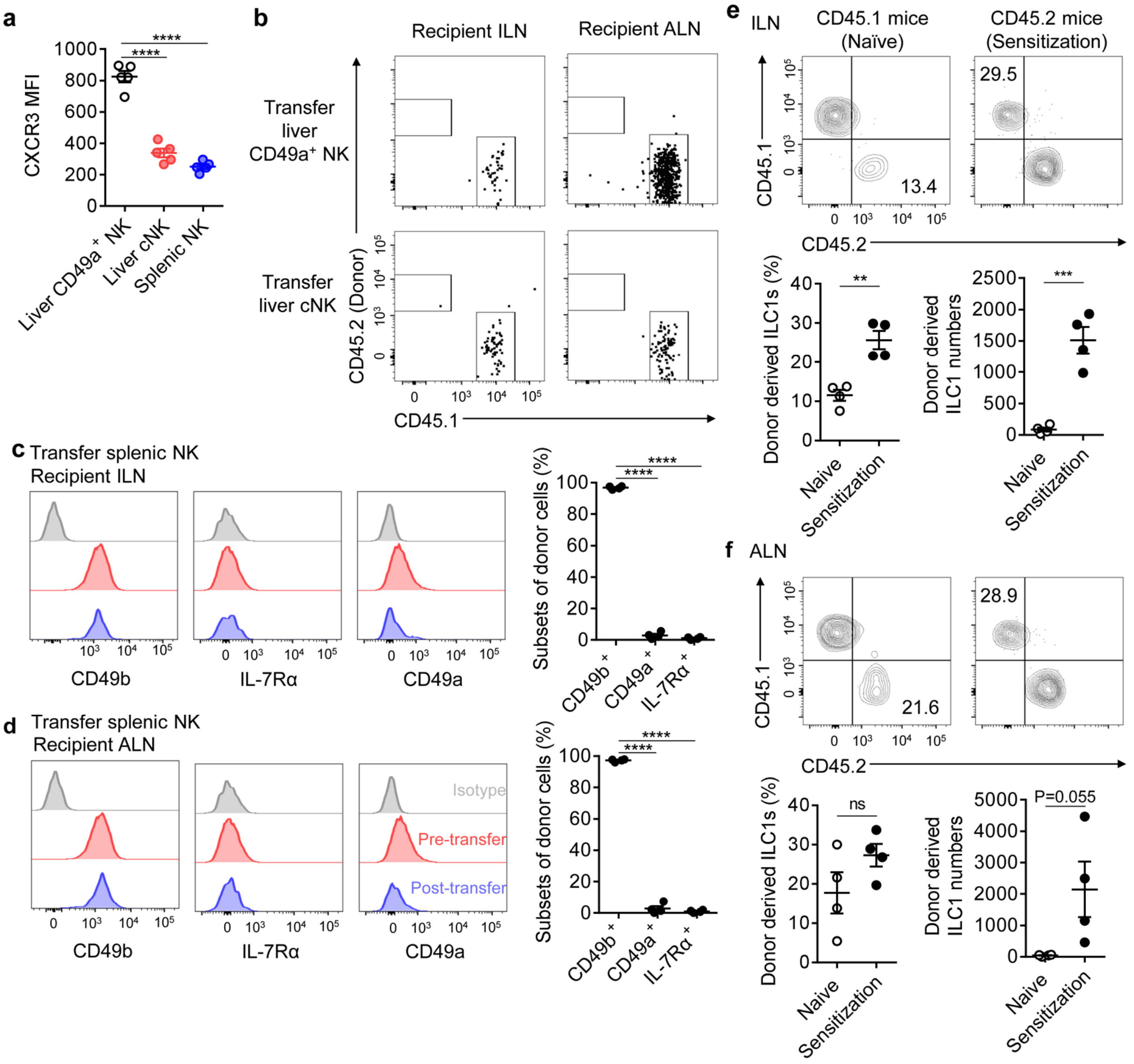

Supplementary Figure 5. Source of hapten-primed ILC1s in LNs.

(**a**) MFI of CXCR3 on liver CD49a$^+$ NK cells, cNK cells, and bulk splenic NK cells. Data are representative of at least three experiments (n = 3–5 in each experiment). (**b**) Liver cNK cells or CD49a$^+$ NK cells (CD45.2$^+$) (3 × 10^5 each) were adoptively transferred into CD45.1 mice. Recipient mice were sensitized with OXA on days 0 and 1. Donor cells were analyzed on day 2. Dot plots are of cells gated on CD3$^-$NK1.1$^+$. Data are representative of two experiments (n = 2 in each experiment). (**c, d**) Phenotype of transferred hapten-primed splenic cNK cells. Naïve splenic cNK cells (CD45.2$^+$) (2 × 10^5) were i.v. transferred to naïve CD45.1 mice, which were then sensitized with OXA. Donor cells (CD45.2$^+$CD3$^-$NK1.1$^+$) in recipient ILNs (**c**) and ALNs (**d**) were

assessed 48 h later. Data are representative of two experiments (n = 4 in each experiment). (**e, f**) Blood-derived LN ILC1s were identified in parabiotic mice. CD45.1 and CD45.2 mice were parabiosed and 2 weeks later, CD45.2 mice were sensitized with OXA, and LN ILC1s were assessed after 48 h. Representative density plots showing CD45.1 and CD45.2 expression of $CD3^-$ $NK1.1^+IL\text{-}7R\alpha^+$ ILC1s in ILNs (**e**) and ALNs (**f**). $CD45.2^+$ ILC1s in CD45.1 mice (naive), and $CD45.1^+$ ILC1s in CD45.2 mice (sensitized) were subject to statistical analysis. Data are pooled from two experiments (n = 4 pairs). Means ± SEM are shown. $^{**}P < 0.01$, $^{***}P < 0.001$, $^{****}P < 0.0001$, two-tailed unpaired Student's *t*-test.

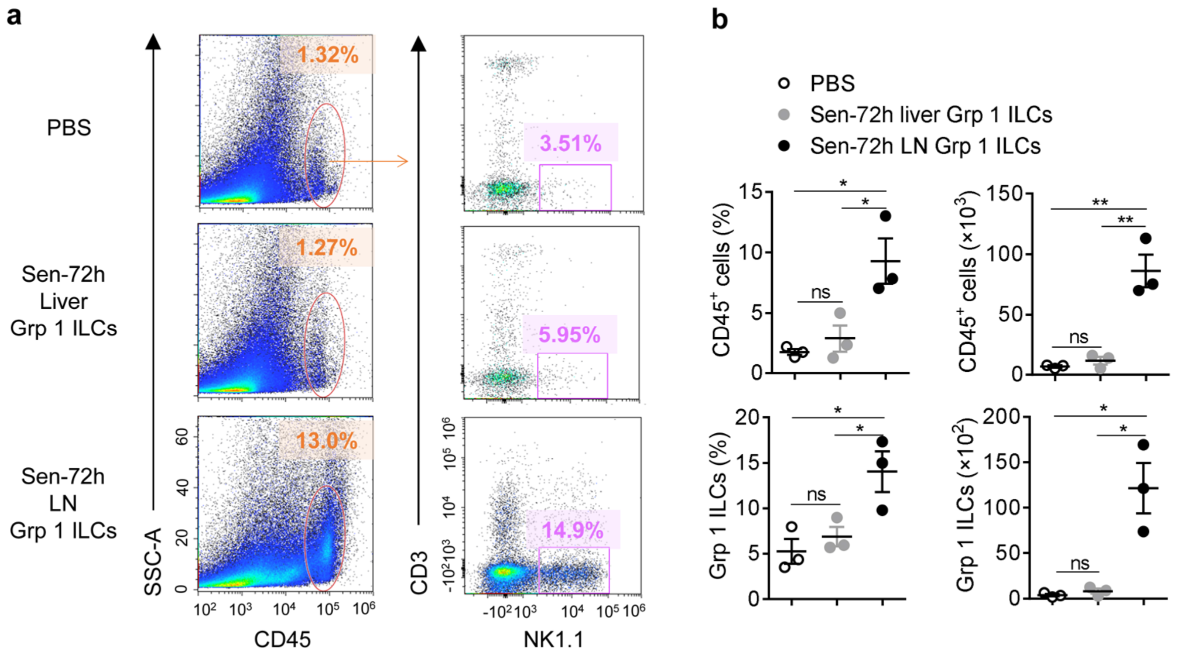

Supplementary Figure 6. Group 1 ILCs accumulate at effector sites.

(**a, b**) Representative dot plots (**a**) showing skin CD45$^+$ cells and group 1 ILCs in B6 mice that received 2 × 10^5 sensitized (72 h) LN or liver group 1 ILCs and were challenged 24 h later. Statistical analysis of results (**b**) showing the percentages and absolute numbers of skin CD45$^+$ cells and group 1 ILCs. Data are pooled from two experiments (n = 3 in each group). Means ± SEM are shown. *$P < 0.05$, **$P < 0.01$, two-tailed unpaired Student's *t*-test.

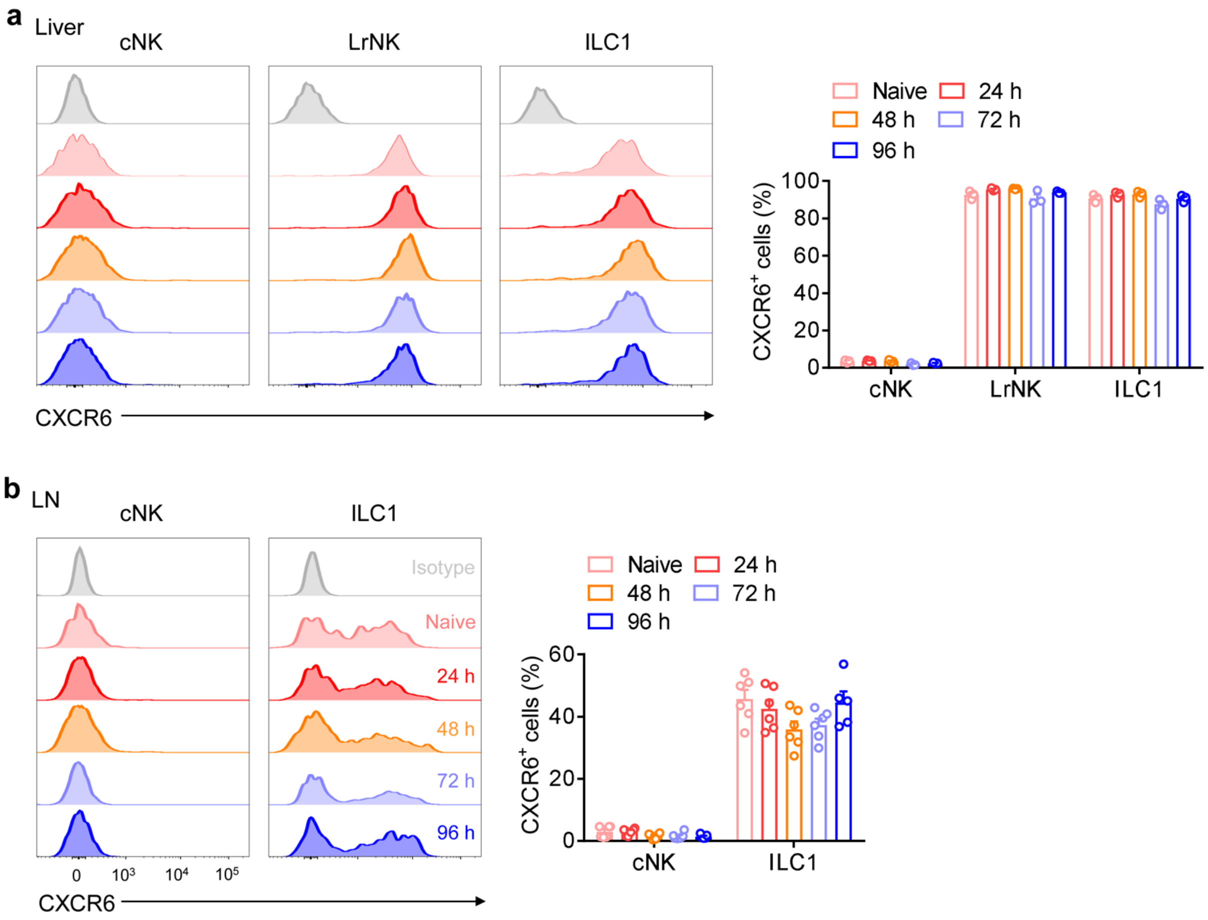

Supplementary Figure 7. CXCR6 expression on cNK cells and ILC1s after hapten sensitization.

(**a, b**) B6 mice were sensitized at different time points. The expression of CXCR6 on liver (**a**) and LN (**b**) group 1 ILC subsets was assessed. Data are pooled from two experiments (n = 6).

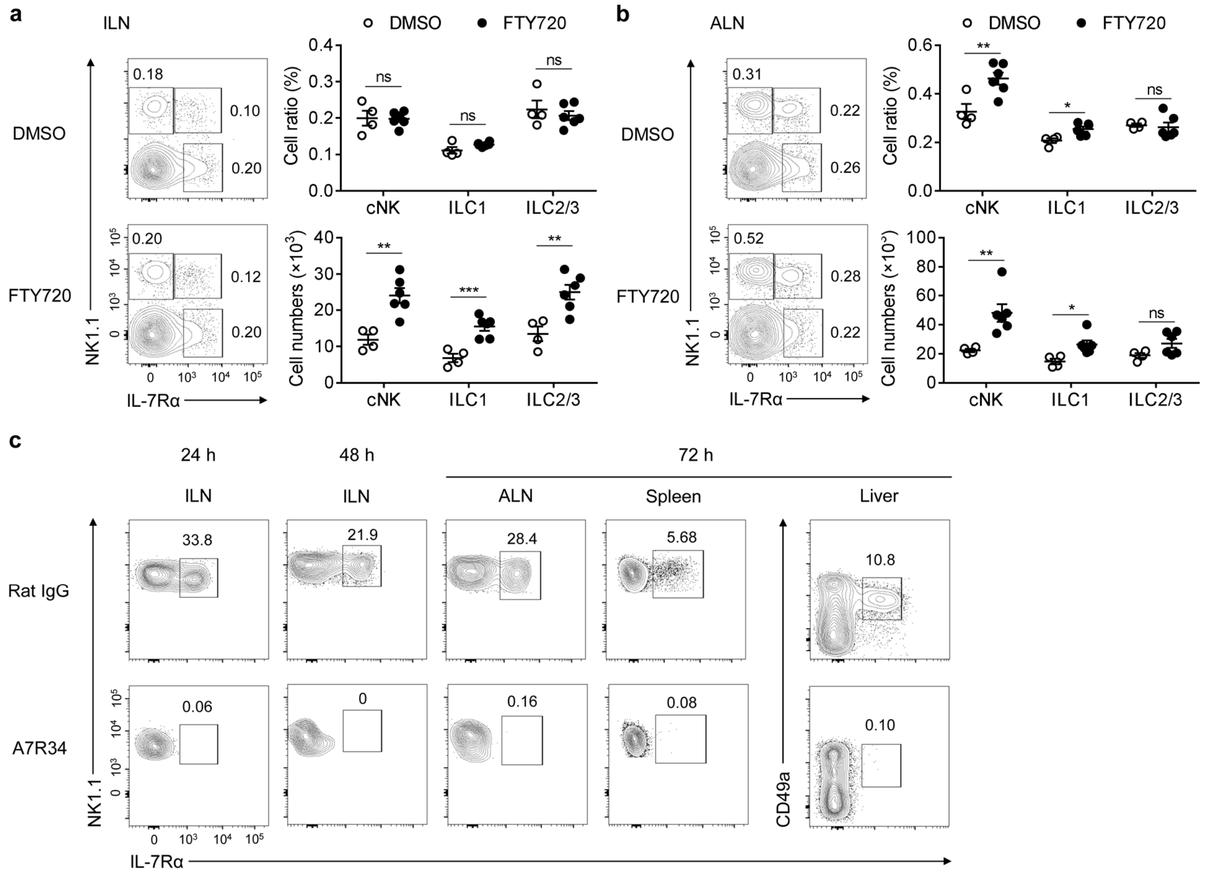

Supplementary Figure 8. In vivo blockade of S1PR1 and IL-7Rα.

(**a**) WT B6 mice were sensitized (day 0, 1) with OXA and i.p. injected with FTY720 daily for 6 consecutive days (days 2–7). On day 8, ILC subsets in ILNs (**a**) and ALNs (**b**) were analyzed. Representative density plots (left panels) showing NK1.1 and IL-7Rα expression on $CD3^{-}CD19^{-}$ cells. Statistical analysis of results (right panels) showing the percentages and absolute numbers of ILC subsets (n = 4–6 in each group). (**c**) On day 0, *Rag1*$^{-/-}$ mice were i.p. injected with 200 µg A7R34 antibody. On days 1, 2, and 3, IL-7Rα expression on ILC1s was analyzed. Representative density plots showing NK1.1/CD49a versus IL-7Rα expression on $CD45^{+}CD3^{-}NK1.1^{+}$ cells in indicated tissues. Data are representative of two independent experiments. Means ± SEM are shown. $*P < 0.05$, $**P < 0.01$, $***P < 0.001$, two-tailed unpaired Student's *t*-test.

Supplementary Table 1. Anti-mouse antibodies for Flow Cytometry.

Antibody	Clone	Company	Dilution
anti-CD3	145-2C11	BioLegend	1:200
anti-NK1.1	PK136	BioLegend	1:200
anti-CD127	A7R34	BioLegend	1:200
anti-CXCR3	CXCR3-173	BioLegend	1:200
anti-CXCR6	SA051D1	BioLegend	1:200
anti-CD19	6D5	BioLegend	1:200
anti-CD45.2	104	BioLegend	1:200
anti-CD45.1	A20	BioLegend	1:200
anti-Thy-1.2	30-H12	BioLegend	1:200
anti-TNF-α	MP6-XT22	BioLegend	1:200
anti-IFN-γ	XMG1.2	BioLegend	1:200
anti-CD45	30-F11	BD Biosciences	1:200
anti-CD49b	DX5	BD Biosciences	1:200
anti-CD49a	Ha31/8	BD Biosciences	1:200
anti-CD62L	MEL-14	BD Biosciences	1:200
anti-CD18	C71/16	BD Biosciences	1:200
anti-Ly49C/I	5E6	BD Biosciences	1:200
anti-T-bet	4-B10	BD Biosciences	1:200
anti-Ly49A	A1	BD Biosciences	1:200
antiLy49G2	4D11	BD Biosciences	1:200
anti-NKp46	29A1.4	BD Biosciences	1:200
anti-Ki67	SolA15	eBioscience	1:200
anti-Eomes	Dan11mag	eBioscience	1:200
anti-NKG2D	CX5	eBioscience	1:200
anti-NKG2A	16a11	eBioscience	1:200
anti-Ly49E/F	CM4	eBioscience	1:200
anti-Ly49H	3D10	eBioscience	1:200
